# Sex Differences in Lung Imaging and SARS-CoV-2 Antibody Responses in a COVID-19 Golden Syrian Hamster Model

**DOI:** 10.1128/mBio.00974-21

**Published:** 2021-07-13

**Authors:** Santosh Dhakal, Camilo A. Ruiz-Bedoya, Ruifeng Zhou, Patrick S. Creisher, Jason S. Villano, Kirsten Littlefield, Jennie Ruelas Castillo, Paula Marinho, Anne E. Jedlicka, Alvaro A. Ordonez, Melissa Bahr, Natalia Majewska, Michael J. Betenbaugh, Kelly Flavahan, Alice R. L. Mueller, Monika M. Looney, Darla Quijada, Filipa Mota, Sarah E. Beck, Jacqueline Brockhurst, Alicia M. Braxton, Natalie Castell, Mitchel Stover, Franco R. D’Alessio, Kelly A. Metcalf Pate, Petros C. Karakousis, Joseph L. Mankowski, Andrew Pekosz, Sanjay K. Jain, Sabra L. Klein

**Affiliations:** a W. Harry Feinstone Department of Molecular Microbiology and Immunology, The Johns Hopkins Bloomberg School of Public Health, Baltimore, Maryland, USA; b Department of Pediatrics, The Johns Hopkins University grid.21107.35grid.471401.7grid.21107.35grid.471401.7grid.21107.35grid.471401.7 School of Medicine, Baltimore, Maryland, USA; c Department of Molecular and Comparative Pathobiology, The Johns Hopkins School of Medicine, Baltimore, Maryland, USA; d Department of Medicine, The Johns Hopkins School of Medicine, Baltimore, Maryland, USA; e Advanced Mammalian Biomanufacturing Innovation Center, Department of Chemical and Biomolecular Engineering, Johns Hopkins University grid.21107.35grid.471401.7grid.21107.35grid.471401.7grid.21107.35grid.471401.7 , Baltimore, Maryland, USA; Duke University School of Medicine

**Keywords:** animal model, COVID-19, sex differences, SARS-CoV-2 variants, receptor-binding domain

## Abstract

In the coronavirus disease 2019 (COVID-19) pandemic caused by the severe acute respiratory syndrome coronavirus 2 (SARS-CoV-2), more severe outcomes are reported in males than in females, including hospitalizations and deaths. Animal models can provide an opportunity to mechanistically interrogate causes of sex differences in the pathogenesis of SARS-CoV-2. Adult male and female golden Syrian hamsters (8 to 10 weeks of age) were inoculated intranasally with 10^5^ 50% tissue culture infective dose (TCID_50_) of SARS-CoV-2/USA-WA1/2020 and euthanized at several time points during the acute (i.e., virus actively replicating) and recovery (i.e., after the infectious virus has been cleared) phases of infection. There was no mortality, but infected male hamsters experienced greater morbidity, losing a greater percentage of body mass, developed more extensive pneumonia as noted on chest computed tomography, and recovered more slowly than females. Treatment of male hamsters with estradiol did not alter pulmonary damage. Virus titers in respiratory tissues, including nasal turbinates, trachea, and lungs, and pulmonary cytokine concentrations, including interferon-β (IFN-β) and tumor necrosis factor-α (TNF-α), were comparable between the sexes. However, during the recovery phase of infection, females mounted 2-fold greater IgM, IgG, and IgA responses against the receptor-binding domain of the spike protein (S-RBD) in both plasma and respiratory tissues. Female hamsters also had significantly greater IgG antibodies against whole-inactivated SARS-CoV-2 and mutant S-RBDs as well as virus-neutralizing antibodies in plasma. The development of an animal model to study COVID-19 sex differences will allow for a greater mechanistic understanding of the SARS-CoV-2-associated sex differences seen in the human population.

## INTRODUCTION

At the start of the coronavirus disease 2019 (COVID-19) pandemic, early publications from Wuhan, China (1, 2), and European countries (3) began reporting male biases in hospitalization, intensive care unit (ICU) admissions, and mortality rates. Ongoing real-time surveillance (4) and meta-analyses of over 3 million cases of COVID-19 (5) continue to show that while the incidence of COVID-19 cases is similar between the sexes, adult males are almost 3 times more likely to be admitted into ICUs and twice as likely to die as females. Differential exposure to the severe acute respiratory syndrome coronavirus 2 (SARS-CoV-2) is likely associated with behaviors, occupations, comorbidities, and societal and cultural norms (i.e., gender differences) that impact the probability of exposure, access to testing, utilization of health care, and risk of disease (6–8). This is distinct but also complementary to biological sex differences (i.e., sex chromosome complement, reproductive tissues, and sex steroid hormone concentrations) that can also impact susceptibility and outcomes from COVID-19 (9, 10). While exposure to SARS-CoV-2 may differ based on gender, the increased mortality rate among males in diverse countries and at diverse ages likely reflects biological sex. Studies have shown that in males, mutations in X-linked genes (e.g., *TLR7*) resulting in reduced interferon (IFN) signaling (11), elevated proinflammatory cytokine production (e.g., interleukin-6 [IL-6] and C-reactive protein [CRP]) (2, 12), reduced CD8^+^ T cell activity (e.g., IFN-γ) (13), and greater antibody responses (i.e., anti-SARS-CoV-2 antigen-specific IgM, IgG, and IgA and neutralizing antibodies) (14) are associated with more severe COVID-19 outcomes than in females. Because COVID-19 outcomes can be impacted by both gender and biological sex, consideration of the intersection of these contributors is necessary in human studies (15).

Animal models can mechanistically explore sex differences in the pathogenesis of SARS-CoV-2 independent of confounding gender-associated factors that impact exposure, testing, and use of health care globally. Transgenic mice expressing human angiotensin converting enzyme 2 (ACE2) (K18-hACE2) are susceptible to SARS-CoV-2, and in this model, males experience greater morbidity than females, despite having similar viral loads in respiratory tissues (e.g., nasal turbinates, trachea, and lungs) (16, 17). Transcriptional analyses of lung tissue revealed that inflammatory cytokine and chemokine gene expression is greater in males than in females early during infection, and these transcriptional patterns show a stronger correlation with disease outcomes among males than females (16, 17). In addition to utilizing hACE2 mice, mouse-adapted strains of SARS-CoV-2 have been developed and can productively infect wild-type mice but have not yet been used to evaluate sex-specific differences in the pathogenesis of disease (18–20).

Golden Syrian hamsters are also being used as an animal model of SARS-CoV-2 pathogenesis because they are susceptible to human strains of viruses, without the need for genetic modifications in either the host or virus. While studies have included males and females in analyses of age-associated differences in the pathogenesis of SARS-CoV-2 (21), few studies have specifically evaluated males versus females to better understand sex differences in disease. There are studies of golden Syrian hamsters that have included male and female hamsters, but they did not have sufficient numbers of animals to accurately compare the sexes (22). Sex differences are not reported in viral RNA, infectious virus, or cytokine mRNA expression at a single time point (i.e., 4 days postinfection) in the lungs of golden Syrian hamsters (23). There is a gap in the literature of studies designed to rigorously test the hypothesis that biological sex alters disease severity and immune responses after SARS-CoV-2 infection.

## RESULTS

### Males experience greater morbidity than females following SARS-CoV-2 infection, which cannot be reversed by estradiol treatment.

**FIG 1:**
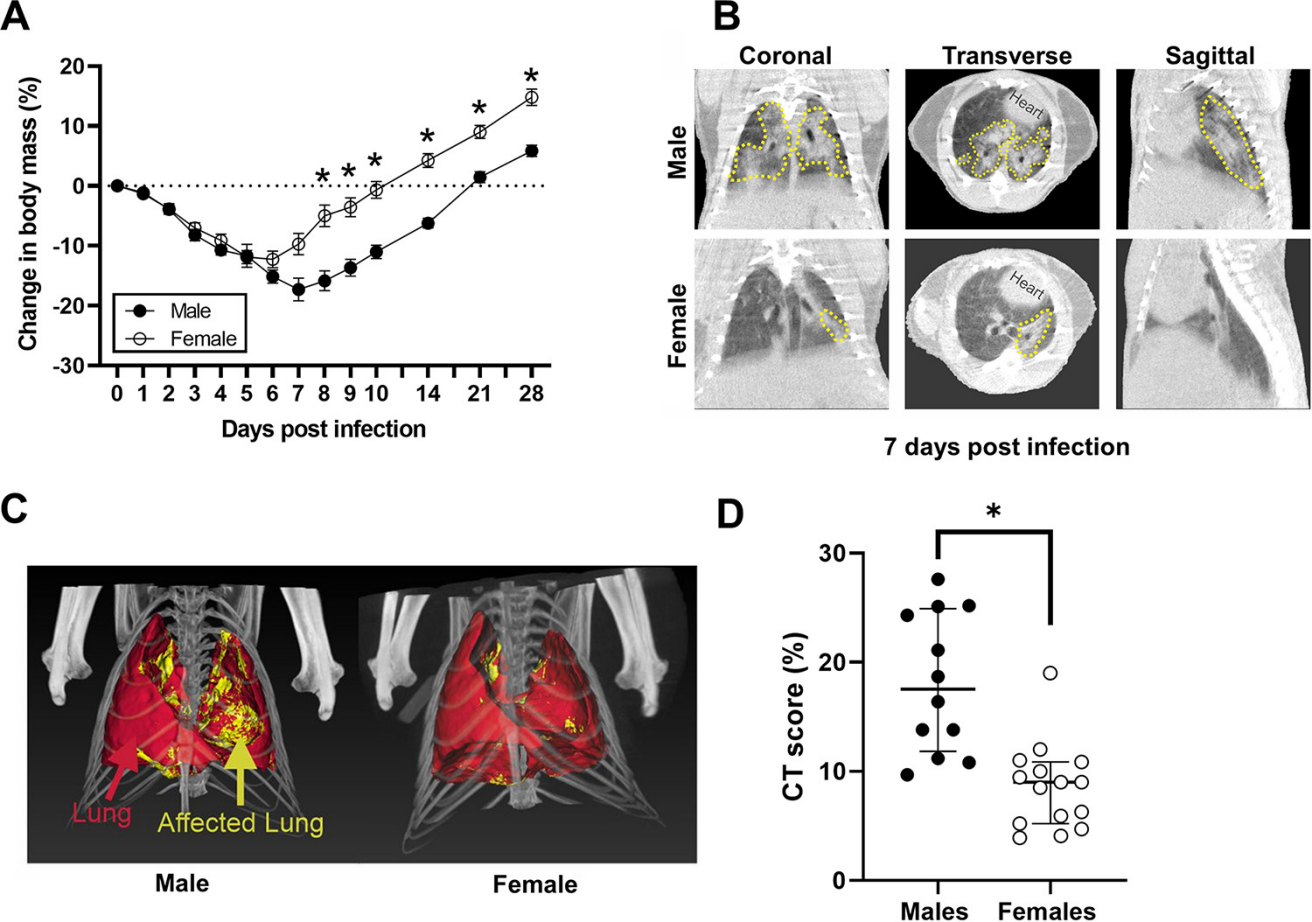
SARS-CoV-2-infected male hamsters experience greater disease than females. (A) To evaluate morbidity, the percent change in body mass from preinoculation was measured up to 28 dpi. (B) Representative coronal, transverse, and sagittal chest CT images from SARS-CoV-2-infected male and female animals are shown. Lung lesions (GGO, consolidation, and air bronchogram) are marked by the dashed yellow lines. (C) Maximum intensity projections (MIP) marking total (red) and diseased lung (yellow) for both males and females are shown. (D) The CT score is higher in male hamsters than in female hamsters at 7 dpi. Weights are represented as mean ± standard error of the mean from two independent replications (*n* = 9 to 10/group), and significant differences between groups are denoted by asterisks (*P < 0.05) based on two-way repeated measures ANOVA followed by Bonferroni’s multiple comparison (A). Chest CT data are represented as median ± interquartile range from two independent replications (*n* = 12 to 15/group), and significance is denoted by an asterisk (*P < 0.05) based on an unpaired two-tailed Mann-Whitney test (D).

Intranasal inoculation of human clinical isolates of SARS-CoV-2 causes productive infection in golden Syrian hamsters (24–26). To test the hypothesis that SARS-CoV-2 infection results in sex differences in disease outcomes, adult male and female golden Syrian hamsters were infected with 10^5^ 50% tissue culture infective dose (TCID_50_) of virus, and changes in body mass were monitored for 28 days postinoculation (dpi). Mortality was not observed in either sex, but infected hamsters progressively lost body mass during the first week before starting to recover (Fig. 1A). The peak body mass loss in female hamsters was observed at 6 dpi (−12.3 ± 1.8%), whereas peak body mass loss in male hamsters was observed at 7 dpi (−17.3 ± 1.9%). The percentage of body mass loss was significantly greater in male than in female hamsters at 8 to 10 dpi and throughout the recovery period (*P* < 0.05; Fig. 1A). Recovery to baseline body mass after SARS-CoV-2 infection occurred within 2 weeks for female hamsters and at 3 weeks for male hamsters (Fig. 1A).

To evaluate pulmonary disease in SARS-CoV-2-infected males and females, chest computed tomography (CT) was performed at the peak of lung disease (7 dpi). As previously reported by others (26), multiple and bilateral mixed ground-glass opacities (GGO) and consolidations were detected in both females and males (Fig. 1B; see also Fig. S1 in the supplemental material). In order to reduce bias in the visual assessment, we developed an unbiased approach to quantify lung disease by chest CT. Volumes of interest (VOIs) were drawn to capture total and diseased (pneumonic) lung volumes (Fig. 1C). As reported in COVID-19 patients who underwent CT (27, 28), there was significantly more disease in the lungs of male hamsters than in female hamsters (*P* < 0.05) (Fig. 1D). These results indicate that infected male hamsters developed more severe disease, including more extensive lung injury, than females.

10.1128/mBio.00974-21.1 FIG S1 Representative transverse chest CT of five female, placebo-treated male, and E2-treated male hamsters at 7 dpi. Multiple bilateral and peripheric ground-glass opacities (GGO) and mixed GGO with consolidations are the hallmark findings at the peak of lung disease. Download FIG S1, TIF file, 2.6 MB. Copyright © 2021 Dhakal et al. 2021 Dhakal et al. https://creativecommons.org/licenses/by/4.0/ This content is distributed under the terms of the Creative Commons Attribution 4.0 International license.

**FIG 2:**
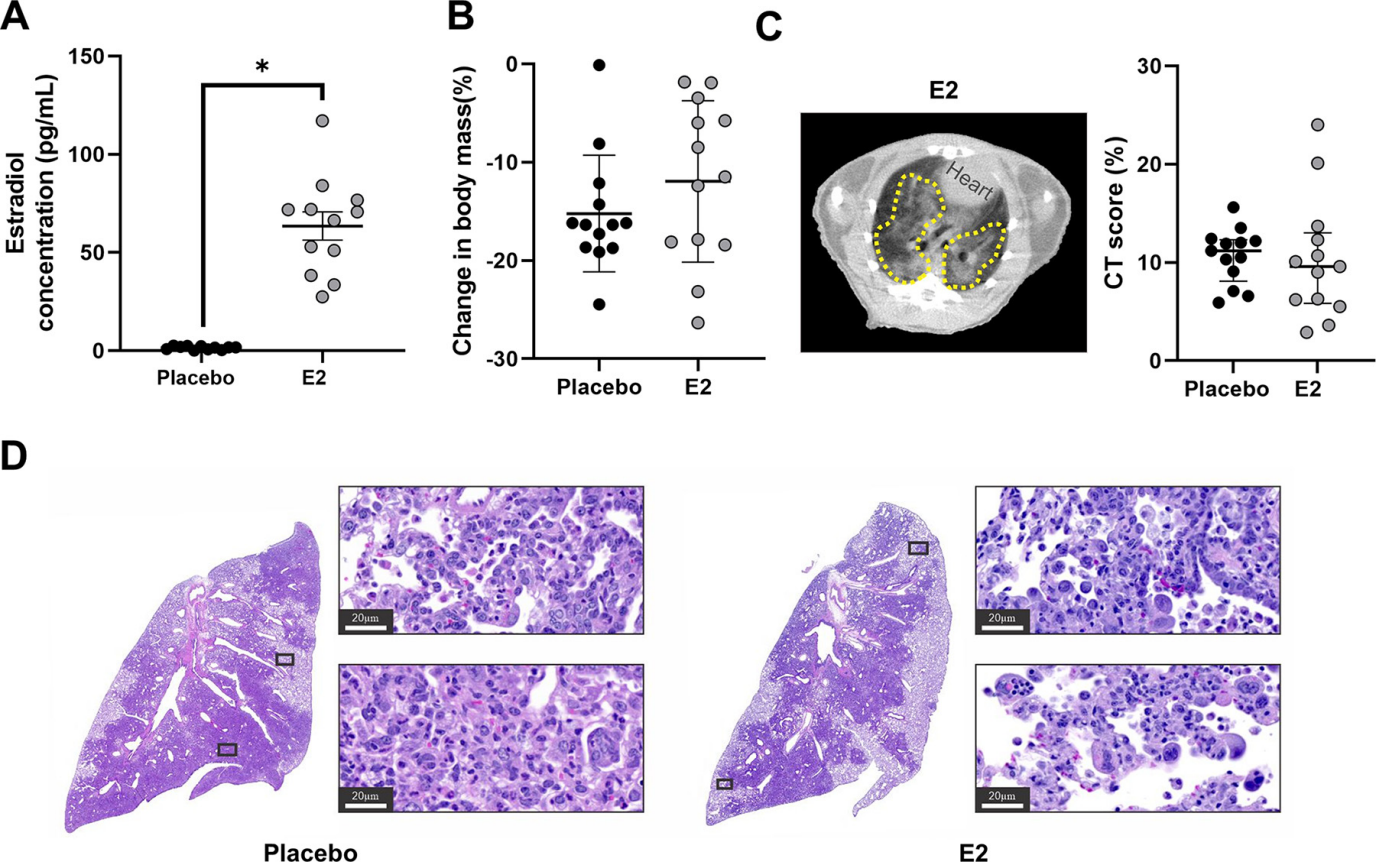
SARS-CoV-2-infected male hamsters treated with estradiol (E2) developed similar lung pathology as placebo-treated males. Male hamsters were treated with E2 capsules or placebo capsules prior to SARS-CoV-2 infection. Estrogen levels were quantified in plasma at 7 dpi (A). Changes in body mass for E2- and placebo-treated males were quantified (B). (C) CT score shows no difference between E2-treated males and placebo-treated males. The dashed yellow lines indicate lung lesions (GGO, consolidations, and air bronchogram). (D) Histopathology (hematoxylin and eosin) data in representative SARS-CoV-2-infected placebo-treated male and E2-treated male hamster lungs at ×40 magnification are shown; scale bars, 20 μm. E2 concentrations are represented as mean ± standard error of the mean of two independent experiments (*n* = 11 to 12/group), and significance is denoted by an asterisk (*P < 0.05) based on two-tailed unpaired *t* test (A). Body mass data are represented as mean ± standard error of the mean of two independent experiments (*n* = 13/group) (B). Chest CT data are represented as median ± interquartile range (IQ) from two independent experiments (*n* = 13/group) (C).

Previous studies show that estrogens, including but not limited to estradiol (E2), are anti-inflammatory and can reduce pulmonary tissue damage following respiratory infections, including with influenza A viruses or Streptococcus pneumoniae (29–31). To test the hypothesis that E2 could dampen inflammation and pulmonary tissue damage to improve outcomes in male hamsters, males received either exogenous E2 capsules or placebo capsules prior to SARS-CoV-2 infection. Plasma concentrations of E2 were significantly elevated in E2-treated males compared with placebo-treated males (*P* < 0.05; Fig. 2A) and were well within the normal range of plasma concentrations of E2 in cyclic female hamsters (30 to 700 pg/ml) (32). Animals were followed for 7 dpi, and changes in body mass and chest CT score were quantified. There was no effect of E2 treatment on morbidity, as placebo- and E2-treated males had equivalent percentages of body mass loss (Fig. 2B). CT findings noted in E2-treated males were similar to those noted in placebo-treated males (Fig. S1), and chest CT scans revealed no difference in CT score between groups (Fig. 2C). At 7 dpi, the lung parenchyma contained consolidation areas consisting of scattered multinucleated syncytia, atypical hyperplastic proliferative type II pneumocytes, rich clusters of macrophages, and vasculitis (Fig. 2D). Moreover, histopathology demonstrated similar cell infiltration and pneumonic areas between groups. From these data, we conclude that the treatment of gonadally intact males with E2 did not improve morbidity or pulmonary outcomes from SARS-CoV-2 infection.

### SARS-CoV-2 replication kinetics are similar between the sexes.

**FIG 3:**
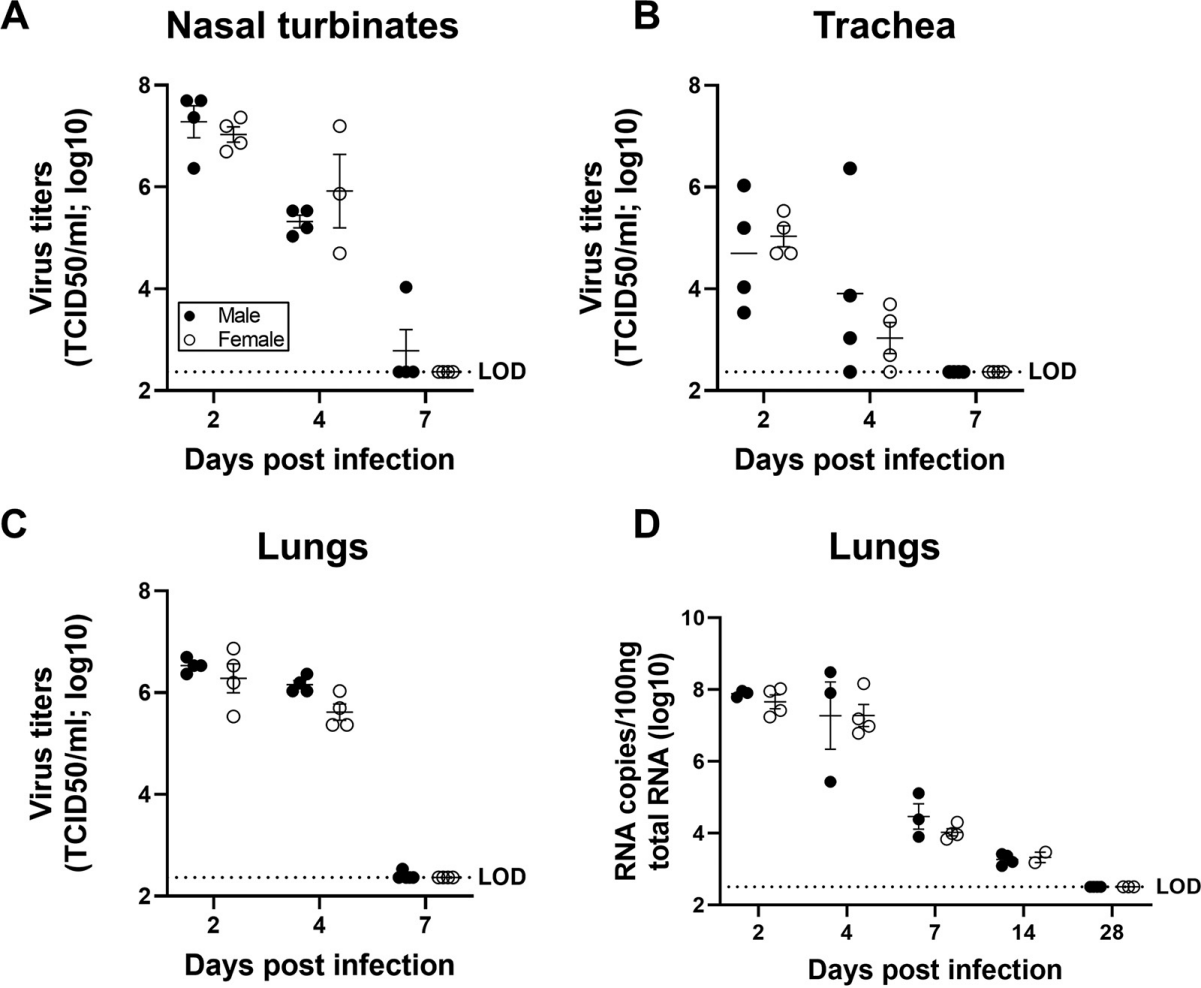
Virus titers were comparable in the respiratory system of SARS-CoV-2-infected male and female hamsters. Adult (8 to 10 weeks) male and female golden Syrian hamsters were infected with 10^5^ TCID_50_ of SARS-CoV-2. Infectious virus titers in the homogenates of nasal turbinates (A), trachea (B), and lungs (C), were determined by TCID_50_ assay on 2, 4, and 7 dpi. Likewise, virus RNA copies in 100 ng of total RNA were assessed in the lungs of infected hamsters at 2, 4, 7, 14 and 28 dpi (D). Data are represented as mean ± standard error of the mean from one or two experiment(s) (*n* = 3 to 5/group) and were analyzed by two-way ANOVA (mixed-effects analysis) followed by Bonferroni’s multiple-comparison test. LOD, limit of detection.

To test the hypothesis that male-biased disease outcomes were caused by increased virus load or faster replication kinetics, subsets of infected male and female hamsters were euthanized at 2, 4, or 7 dpi, and infectious virus titers were measured in the respiratory tissue homogenates. The peak infectious virus titers in the nasal turbinates (Fig. 3A), trachea (Fig. 3B), and lungs (Fig. 3C) were detected at 2 dpi, decreased at 4 dpi, and cleared at 7 dpi. There were no sex differences in either peak virus titers or clearance of SARS-CoV-2 from any of the respiratory tissues tested (Fig. 3A to C). Although the infectious virus was cleared from the respiratory tract of most of the hamsters by 7 dpi (Fig. 3A to C), viral RNA was still detectable in the lungs at 14 dpi in all of the SARS-CoV-2-infected hamsters, with no differences between the sexes (Fig. 3D). These data illustrate that sex differences in the disease phenotype are not due to differences in infectious virus loads or persistence of viral RNA.

### Cytokine concentrations in the lungs are comparable between the sexes.

**FIG 4:**
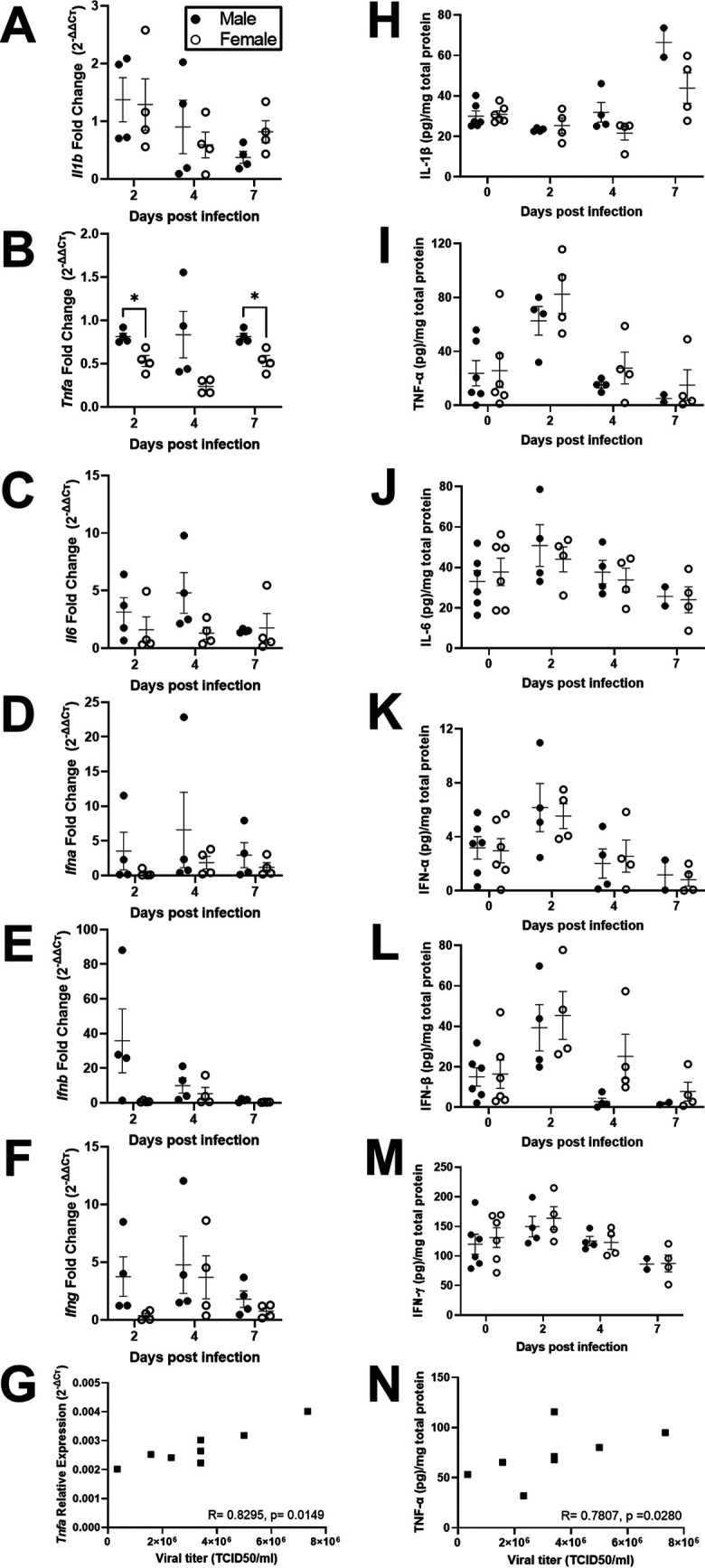
Cytokine responses in the lungs of SARS-CoV-2-infected male and female hamsters were comparable. Adult (8 to 10 weeks) male and female golden Syrian hamsters were infected with 10^5^ TCID_50_ of SARS-CoV-2. Subsets of animals were euthanized at different dpi, and IL-1β (A, H), TNF-α (B, I), IL-6 (C, J), IFN-α (D, K), IFN-β (E, L), and IFN-γ (F, M) mRNA expression (fold change as determined by the threshold cycle [ΔΔ*C_T_*] method; A to G) and cytokine concentrations (pg/mg total protein; H to M) were determined in the lungs by reverse transcription qPCR or ELISA. Mock-infected animal samples from different dpi were presented together as 0 dpi for protein concentration. Associations between relative expression (Δ*C_T_* method; G) and concentration (pg/mg total protein; N) of TNF-α and virus titers in lungs collected 2 dpi were analyzed by Spearman correlation analyses, with a significant association represented with the *R* statistic and associated *P* value. Data represent mean ± standard error of the mean from one or two independent experiments (*n* = 2 to 6/group/sex) and were analyzed by two-way ANOVA (mixed-effects analysis) followed by Bonferroni’s multiple-comparison test.

To test whether local or systemic cytokine activity differed between the sexes, cytokine mRNA expression was measured in lung tissue, and protein concentrations of cytokines were measured in lung and spleen homogenates at 2, 4, or 7 dpi. Transcriptionally, although males and females had similar pulmonary expression of *Il1b*, *Il6*, *Ifna*, *Ifnb*, and *Ifng*, males had greater expression of *Tnfa* than females (*P* < 0.05; Fig. 4A to F), which was also associated with greater viral titers at 2 dpi in both sexes (*P* < 0.05; Fig. 4G). Sex differences were not observed in the concentrations of IL-1β, TNF-α, IL-6, IFN-α, IFN-β, IFN-γ, or IL-10 in either lung (Fig. 4H to M) or spleen (Table S1) homogenates; concentrations of TNF-α in the lungs, however, were positively associated with virus titers at 2 dpi (*P* < 0.05; Fig. 4N), and concentrations of IFN-β were negatively associated with virus titers at 4 dpi (*R* = −0.85, *P* < 0.05; data not shown) in both sexes. When cytokine data were aggregated for male and female hamsters, there was no consistent pattern of mRNA expression in the lungs (Fig. S2A to F). Lung concentrations of IL-1β (Fig. S2G), TNF-α (Fig. S2H), IFN-α (Fig. S2J), and IFN-β (Fig. S2K), but not IL-6 (Fig. S2I), IFN-γ (Fig. S2L), or IL-10 (Table S1), were greater in samples from infected hamsters than in sex-matched mock-infected hamsters (*P* < 0.05 in each case). There was no effect of infection on the concentration of cytokines in the spleen (Table S1). Taken together, these data provide no evidence that male-biased disease outcomes are caused by differential production of cytokines in response to SARS-CoV-2 during acute infection.

10.1128/mBio.00974-21.2 FIG S2 Kinetics of cytokine concentrations (pg/mg total protein) in the lungs of SARS-CoV-2-infected hamsters. Male and female golden Syrian hamsters were infected with 10^5^ TCID_50_ of SARS-CoV-2. Subsets of animals were euthanized at different dpi, and IL-1β (A, G), TNF-α (B, H), IL-6 (C, I), IFN-α (D, J), IFN-β (E, K), and IFN-γ (F, L) mRNA expression (fold change as determined by the ΔΔ*C_T_* method; A to F) and cytokine concentrations (pg/mg total protein; G to L) were determined in the lungs by reverse transcription qPCR or ELISA. Mock-infected animal samples from 2, 4, or 7 days post infection (dpi) were not statistically different and were combined and presented together as 0 dpi for protein concentration. Data represent mean ± standard error of the mean from one or two independent experiments (*n* = 6 to 12/group), with significant differences between groups denoted by asterisks (*P < 0.05) based on one-way ANOVA followed by Dunnett’s multiple-comparison test. Download FIG S2, TIF file, 0.4 MB. Copyright © 2021 Dhakal et al. 2021 Dhakal et al. https://creativecommons.org/licenses/by/4.0/ This content is distributed under the terms of the Creative Commons Attribution 4.0 International license.

10.1128/mBio.00974-21.3 TABLE S1 Concentrations (pg/mg total protein) of cytokines in the lungs and spleen of male and female hamsters at different days post infection (dpi). Mock-infected animal samples from different dpi were pooled and used as 0 dpi. Data are presented as the mean ± standard error of the mean from one or two independent experiments (*n* = 6 to 12/group) with no significant differences observed between the groups based on two-way ANOVA (mixed-effects analysis) followed by Bonferroni’s multiple-comparison test. Download Table S1, PDF file, 0.1 MB. Copyright © 2021 Dhakal et al. 2021 Dhakal et al. https://creativecommons.org/licenses/by/4.0/ This content is distributed under the terms of the Creative Commons Attribution 4.0 International license.

### Female hamsters develop greater antibody responses than males during SARS-CoV-2 infection.

**FIG 5:**
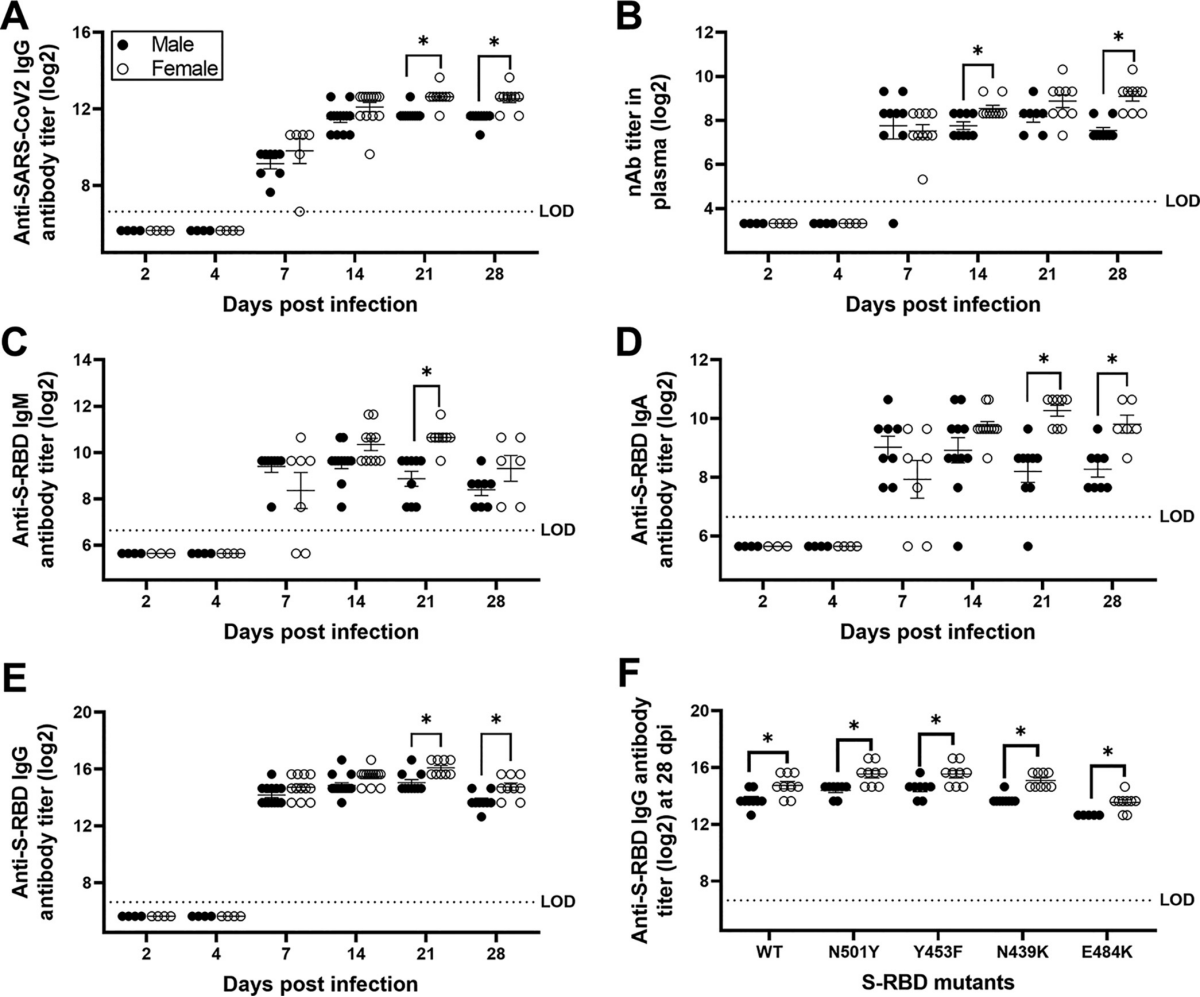
Antibody responses in the plasma of SARS-CoV-2-infected female hamsters were greater than in males. Plasma samples were collected at different dpi, and IgG antibody responses against whole-inactivated SARS-CoV-2 virions (A), virus-neutralizing antibody responses (B), and S-RBD-specific IgM (C), IgA (D), and IgG (E) antibodies were determined. Likewise, cross-reactive IgG antibodies against mutant S-RBDs (*viz.* N501Y, Y453F, N439K, and E484K) were evaluated in plasma at 28 dpi (F). Considering similar antibody responses at 6 and 7 dpi, values were presented together as 7 dpi. Data represent mean ± standard error of the mean from two independent experiments (*n* = 4 to 14/group/sex), and significant differences between groups are denoted by asterisks (*P < 0.05) based on two-way ANOVA (mixed-effects analysis) followed by Bonferroni’s multiple-comparison test.

To evaluate whether females developed greater antiviral antibody responses than males, as is observed in response to influenza A viruses (33), we measured virus-specific immunoglobulins as well as neutralizing antibody (nAb) titers in plasma and respiratory samples collected throughout the course of infection. To begin our evaluation, we inactivated SARS-CoV-2 virions to analyze plasma IgG that recognize diverse virus antigens. Anti-SARS-CoV-2 IgG titers were detected within a week postinfection, with females developing greater antibody titers than males at 21 and 28 dpi (*P* < 0.05; Fig. 5A). Using live SARS-CoV-2, we measured nAb titers in plasma, which were detectable 7 to 28 dpi, with females having or trending toward significantly greater titers than males at 14 to 28 dpi (*P* < 0.05; Fig. 5B).

SARS-CoV-2 infection induces robust antibody responses against the spike protein or receptor-binding domain (RBD) of the spike protein (S-RBD) in humans and in animal models (14, 34, 35). S-RBD-specific IgM (Fig. 5C), IgA (Fig. 5D), and IgG (Fig. 5E) antibodies were detected in plasma within a week postinfection. In plasma, anti-S-RBD IgM antibody titers were significantly greater in females than in males at 21 dpi (*P* < 0.05; Fig. 5C), and anti-S-RBD IgA and IgG antibody titers were significantly greater in females than in males at 21 and 28 dpi (*P* < 0.05; Fig. 5D and E). Variants of SARS-CoV-2 due to mutations in the RBD of the spike protein, including the N501Y variant, were first reported in the United Kingdom and subsequently circulated worldwide (36). The mink variant (Y453F), European variant (N439K), and South African/Brazilian variants (E484K) have raised concerns over increased transmissibility and escape from host immune responses (37, 38). Considering the emergence of novel variants, we tested the hypothesis that females would have greater cross-reactive antibody responses to SARS-CoV-2 variants. Similar to wild-type S-RDB (Fig. 5E), IgG antibody titers against the S-RBD mutants N501Y, Y453F, N439K, and E484K were significantly greater in female hamsters than in male hamsters (*P* of <0.05 in each case; Fig. 5F). Overall, IgG responses to the E484K variant, but not the N501Y variant, were significantly lower in both sexes than responses to the wild-type S-RBD (*P* of <0.05 for main effect of variant; Fig. 5F).

**FIG 6:**
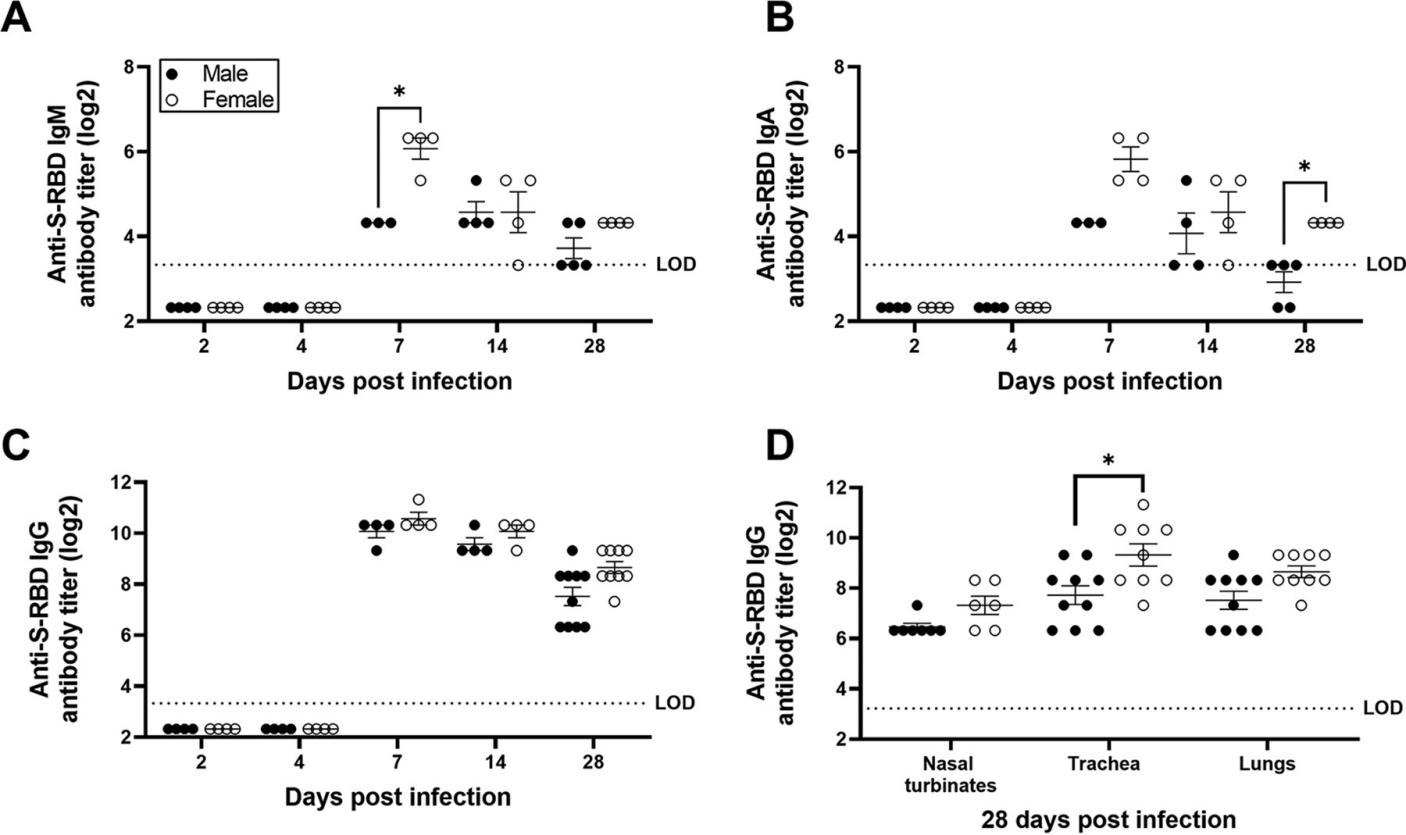
Antibody responses in the respiratory system of SARS-CoV-2-infected female hamsters were greater than in males. Lung homogenates were prepared at different dpi, and S-RBD-specific IgM (A), IgA (B), and IgG (C) antibodies were determined. Likewise, S-RBD-specific IgG antibodies were tested in the homogenates of nasal turbinates, trachea, and lungs at 28 dpi (D). Data represent mean ± standard error of the mean from one or two independent experiment(s) (*n* = 3 to 10/group), and significant differences between groups are denoted by asterisks (*P < 0.05) based on two-way ANOVA (mixed-effects analysis) followed by Bonferroni’s multiple-comparison test.

Local antibody responses at the site of infection are critical for SARS-CoV-2 control and recovery (39, 40). Anti-S-RBD IgM titers were greatest in the lungs at 7 dpi and were significantly greater in female hamsters than in male hamsters (*P* < 0.05; Fig. 6A). A cornerstone of mucosal humoral immunity is IgA, and anti-S-RBD IgA titers peaked at 7 dpi, with a trend for higher titers in females than in males (*P* = 0.07; Fig. 6B). By 28 dpi, females still had detectable anti-S-RBD IgA titers in their lungs, whereas males did not (*P* < 0.05; Fig. 6B). Anti-S-RBD IgG titers in the lungs were elevated 7 to 28 dpi, with a higher trend observed at 28 dpi in females than in males (*P* = 0.09; Fig. 6C). In the trachea, but not in nasal turbinate or lung homogenates, females had significantly greater anti-S-RBD IgG titers than males (*P* < 0.05; Fig. 6D). In summary, these data demonstrate that female hamsters develop greater systemic and local antiviral antibody responses than male hamsters during SARS-CoV-2 infection.

## DISCUSSION

Sex differences in COVID-19 outcomes are well documented (9, 13). There is a critical need to develop accurate animal models that reflect the male bias in disease outcomes to better understand the underlying mechanisms. We show that male hamsters suffer more systemic (body mass loss) and local (pulmonary pathology) symptoms of SARS-CoV-2 infection than females. We tested several potential mechanisms that could mediate male-biased outcomes from infection, including (i) lack of estrogenic protection, (ii) greater virus replication, (iii) exacerbated cytokine responses, and (iv) reduced humoral immunity. Our data reveal that females produce greater antibody responses, both locally in the respiratory tract as well as systemically in plasma, but whether this causes female hamsters to suffer less severe outcomes from SARS-CoV-2 infection remains to be determined.

Clinical manifestations of SARS-CoV-2 infection in hamsters are typically mild, with reduced body mass after infection consistently observed (21, 24–26). Previous studies have shown that hamsters lose body mass after infection, reaching peak loss at 5 to 7 dpi followed by recovery (24–26). Body mass loss in hamsters, regardless of age, has been associated with the dose of virus inoculum, with higher dose resulting in greater body mass loss (26, 41). Body mass loss also is influenced by age; older hamsters (i.e., 7 to 9 months old) had greater body mass loss than younger animals (i.e., 4 to 6 weeks old) (21, 26). Sex is another factor impacting body mass loss in hamsters following SARS-CoV-2 infection. As reported in humans, older age and male sex are clinical variables associated with greater clinical manifestations of disease in hamsters.

A novel determinant of clinical disease that was utilized in the current study was unbiased, quantitative chest CT-imaging analysis. Previous reports describe chest CT findings in female SARS-CoV-2-infected hamsters only and show lung abnormalities, including multilobular ground-glass opacities (GGO) and consolidation (26), as observed in patients with COVID-19 (42). In the current study, CT imaging revealed that multilobular GGO and consolidations were observed to a greater extent in male than in female SARS-CoV-2-infected hamsters at 7 dpi. Whether the sexes differ in the recovery of pulmonary damage following infection requires greater consideration. There are a number of registered clinical trials of therapeutic E2 administration (NCT04359329 and NCT04539626) in COVID-19, which raised the question as to whether disease outcomes in male hamsters could be improved through administration of E2. In this study, pretreatment of male hamsters with E2 prior to SARS-CoV-2 infection did not reduce weight loss, observed histological damage to lung tissue, or the observed multilobular GGO and consolidations.

While male hamsters could not be protected with exogenous E2 treatment, there is still the possibility that testosterone in males is driving more severe disease. A small prospective cohort study in human COVID-19 patients showed that men with lower testosterone concentrations during hospitalization had greater disease severity and markers of inflammation (43). Because COVID-19 is more severe in older men than in younger men, it also could be that the observation of lower testosterone being associated with greater inflammation and more severe disease is confounded by the average age of the patients being greater than 65 years of age. In studies utilizing young adult mice, testosterone has been shown to be anti-inflammatory and is associated with reduced cytokine concentrations and frequencies of inflammatory monocytes and virus-specific CD8^+^ T cells in the lungs during influenza A virus infection (44, 45). Future studies must further explore the direct effects of testosterone on the pathogenesis of SARS-CoV-2 in golden Syrian hamsters.

SARS-CoV-2 replicates in the nasal turbinates, trachea, and lungs of infected golden Syrian hamsters (24, 25). Virus replication peaks in respiratory tissue within 2 to 4 dpi, with virus clearance typically occurring within 1 week (21, 24, 25). Viral RNA, however, is present in the lungs of infected hamsters beyond 7 dpi (21, 22, 25). We observed peak infectious virus load in nasal turbinates, trachea, and lungs at 2 dpi, with clearance by 7 dpi. After infectious virus had been cleared, viral RNA still remained detectable in the lungs up to 14 dpi. Previous studies have reported that while aged hamsters experience worse disease outcomes than young hamsters, virus titers in respiratory tissues are similar (21, 26). We further show that although young adult male hamsters experience worse disease outcomes than female hamsters, sex differences in virus titers in respiratory tissues are not observed.

During SARS-CoV-2 infection of hamsters, cytokine gene expression, including *Tnfα*, *Ifnα*, and *Ifnγ* in the nasal turbinates and lungs, is triggered at 2 dpi, peaks at 4 dpi, and returns to baseline by 7 dpi, but comparisons between males and females have not performed (24, 41, 46). Analyses of protein concentrations of cytokines in lung and spleen homogenates revealed no differences between males and females during the first week of infection. Although sex differences were not observed, concentrations of TNF-α and IFN-β were positively and negatively, respectively, associated with virus replication in lungs, regardless of sex. Our findings suggest that cytokine production, either locally in the lungs or systemically in the spleen, does not underlie sex differences in clinical manifestations of disease in hamsters and adds to the growing list of questions about the role of cytokines in the pathogenesis of SARS-CoV-2 in human populations. The possibility of differences in cellular infiltration into pulmonary tissue requires greater consideration, which will be feasible only when better reagents, including antibodies, become available for hamsters.

Studies have reported that both IgG and virus-neutralizing antibodies are detected in serum from SARS-CoV-2-infected golden Syrian hamsters as early as 7 dpi and persist through 43 dpi (24, 35, 47). In the present study, females developed greater IgG responses against both SARS-CoV-2 wild-type and variant S-RBD as well as antiviral nAb titers in both plasma and respiratory tissue homogenates than males. We also showed that mucosal IgA titers are greater in the lungs of female hamsters than in male hamsters and are detectable as early as 7 dpi. Passive transfer of convalescent-phase sera from infected to naive hamsters as well as reinfection of previously infected hamsters carrying high antibody titers have both been shown to provide protection by reducing virus titers in the respiratory tissues (24, 26). Likewise, hamster models of SARS-CoV-2 immunization have shown an inverse correlation between antibody responses and either virus titers in the respiratory tissues or body mass loss (48). These studies highlight the possible protective role of antibodies during SARS-CoV-2 infection, which may contribute to faster recovery in female hamsters than in male hamsters.

Golden Syrian hamsters have already been successfully used in SARS-CoV-2 transmission studies (24, 25, 49) to compare routes of SARS-CoV-2 infection (41, 50), to evaluate convalescent plasma and monoclonal antibody therapy (24, 26, 51–53), and to test therapeutics and vaccines (23, 48). This model provides a unique opportunity to understand the kinetics of SARS-CoV-2 immunopathology not only systemically but also at the site of infection. Sex as a biological variable should be considered in all studies utilizing golden Syrian hamsters for prophylactic and therapeutic treatments against SARS-CoV-2.

## MATERIALS AND METHODS

### Viruses, cells, and viral proteins.

Vero-E6-TMPRSS2 cells were cultured in complete cell growth medium (CM) composed of Dulbecco’s modified Eagle medium (DMEM) supplemented with 10% fetal bovine serum (FBS), 1 mM glutamine, 1 mM sodium pyruvate, and penicillin (100 U/ml) and streptomycin (100 μg/ml) antibiotics (54). The SARS-CoV-2 strain (SARS-CoV-2/USA-WA1/2020) was obtained from Biodefense and Emerging Infections Research Resources Repository (NR number 52281; BEI Resources, VA, USA). SARS-CoV-2 stocks were generated by infecting Vero-E6-TMPRSS2 cells at a multiplicity of infection (MOI) of 0.01 TCID_50_s per cell, and the infected cell culture supernatant was collected at 72 h postinfection, clarified by centrifugation at 400 × *g* for 10 min and then stored at −70°C (54). SARS-CoV-2 recombinant spike receptor-binding domain (S-RBD) protein used for enzyme-linked immunosorbent assay (ELISA) was expressed and purified using methods described previously (14) or purchased from SinoBiologicals. To obtain whole-inactivated SARS-CoV-2, Vero-E6-TMPRSS2 cells were infected at an MOI of 0.01, and the infected cell culture supernatant was collected at 72 h postinfection. Virus was inactivated by the addition of 0.05% beta-propiolactone (54) followed by incubation at 4°C for 18 h. The beta-propiolactone was inactivated by incubation at 37°C for 2 h, the inactivated virions were pelleted by ultracentrifugation at 25,000 × *g* for 1 h at 4°C, and protein concentration was determined by bicinchoninic acid (BCA) assay (Thermo Fisher Scientific).

### Animal experiments.

Male and female golden Syrian hamsters (7 to 8 weeks of age) were purchased from Envigo (Haslett, MI). Animals were housed under standard housing conditions (68 to 76°F, 30 to 70% relative humidity, 12-h light/12-h dark cycle) in positive/negative control (PNC) cages (Allentown, NJ) with paper bedding (Teklad 7099 TEK-Fresh, Envigo, Indianapolis, IN) in an animal biological safety level 3 (ABSL-3) facility at the Johns Hopkins University-Koch Cancer Research Building. Animals were given nesting material (Enviropak, Lab Supply, Fort Worth, TX) and *ad libitum* reverse osmosis (RO) water and feed (2018 SX Teklad, Envigo, Madison, WI). After 1 to 2 weeks of acclimation, animals (8 to 10 weeks of age) were inoculated with 10^5^ TCID_50_ of SARS-CoV-2 USA-WA1/2020 in 100 μl of DMEM (50 μl/naris) through the intranasal route under ketamine (60 to 80 mg/kg) and xylazine (4 to 5 mg/kg) anesthesia administered intraperitoneally. Control animals received an equivalent volume of DMEM. Animals were randomly assigned to be euthanized at 2, 4, 7, 14, or 28 days postinfection (dpi). Body mass was measured at the day of inoculation (baseline) and endpoint, with daily measurements up to 10 dpi and on 14, 21, and 28 dpi, when applicable per group. Blood samples were collected preinoculation (baseline) and at days 7, 14, 21, and 28 dpi, when applicable per group. Survival blood collection was performed on the sublingual vein, whereas terminal bleeding was done by cardiac puncture under isoflurane (500-μl drop jar; Fluriso, VetOne, Boise, ID) anesthesia. Blood was collected into EDTA (survival and terminal) and/or sodium citrate tubes (terminal). Plasma was separated by blood centrifugation at 3,500 rpm for 15 min at 4°C. After cardiac puncture, animals were humanely euthanized using a euthanasia solution (Euthasol, Virbac, Fort Worth, TX). Nasal turbinates, trachea, and lung samples for antibody/cytokine assays and virus titration were snap-frozen in liquid nitrogen and stored at −80°C.

### Determination of infectious virus titers and viral genome copies in tissue homogenates.

To obtain tissue homogenates, DMEM with 100 U/ml penicillin and 100 μg/ml streptomycin was added (10% wt/vol) to tubes containing hamster nasal turbinate, lung, and tracheal tissue samples. Lysing Matrix D beads were added to each tube, and the samples were homogenized in a FastPrep-24 benchtop bead beating system (MPBio) for 40 s at 6.0 m/s, followed by centrifugation for 5 min at 10,000 × *g* at room temperature. Samples were returned to ice, and the supernatant was distributed equally into 2 tubes. To inactivate SARS-CoV-2, Triton X-100 was added to one of the tubes to a final concentration of 0.5% and incubated at room temperature for 30 min. The homogenates were stored at −70°C.

Infectious virus titers in respiratory tissue homogenates were determined by TCID_50_ assay (14, 54). Briefly, tissue homogenates were 10-fold serially diluted in infection medium (CM with 2.5% instead of 10% FBS), transferred in sextuplicate into the 96-well plates confluent with Vero-E6-TMPRSS2 cells, incubated at 37°C for 4 days, and stained with naphthol blue black solution for visualization. The infectious virus titers in TCID_50_/ml were determined by the Reed and Muench method. For detection of SARS-CoV-2 genome copies, RNA was extracted from lungs using the Qiagen viral RNA extraction kit (Qiagen), and reverse transcription PCR (RT-qPCR) was performed as described (55).

### Computed tomography and image analysis.

Live animals were imaged inside in-house-developed, sealed biocontainment devices compliant with BSL-3, as previously reported (56). Seven days postinfection, SARS-CoV-2-infected male (*n* = 12), female (*n* = 12), placebo-treated male (*n* = 13), and E2-treated male (*n* = 13) hamsters underwent chest CT using the nanoScan positron emission tomography (PET)/CT (Mediso USA, MA, USA) small animal imager. CT images were visualized and analyzed using the VivoQuant 2020 lung segmentation tool (Invicro, MA, USA) (57). Briefly, an entire lung volume (LV) was created, and volumes of interests (VOIs) were shaped around the pulmonary lesions using global thresholding for Hounsfield Units (HU) ≥ 0, and disease severity (CT score) was quantified as the percentage of diseased lung in each animal. The investigators were blinded to the group assignments.

### Hormone replacement and quantification.

Estradiol (E2) capsules were prepared with Silastic brand medical-grade tubing (0.062 in. inside diameter [i.d.] × 0.125 in. outside diameter [o.d.]) 10 mm in length, sealed with Factor II 6382 room temperature vulcanizing silicone and elastomer and filled 5 mm with 17β-estradiol (58). Capsules were incubated overnight in sterile saline at 37°C prior to implantation. The E2 dosage was chosen because this size capsule has previously been shown to produce blood levels within the physiological range of E2 measured in intact female hamsters during early proestrus (when E2 levels are at their peak) (59, 60). Circulating concentrations of E2 were measured by a rodent estradiol ELISA kit as per manufacturer’s instructions (Calbiotech, CA).

### Antibody ELISAs.

The hamster antibody ELISA protocol was modified from the human COVID-19 antibody ELISA protocol described previously (14). ELISA plates (96-well plates; Immunol 4HBX, Thermo Fisher Scientific) were coated with either spike receptor-binding domain (S-RBD) or whole-inactivated SARS-CoV-2 proteins (2 μg/ml, 50 μl/well) in 1× phosphate-buffered saline (PBS) and incubated at 4°C overnight. Coated plates were washed three times with wash buffer (1× PBS + 0.1% Tween 20), blocked with 3% nonfat milk solution in wash buffer, and incubated at room temperature for 1 h. After incubation, blocking buffer was discarded, 2-fold serially diluted plasma (starting at a 1:100 dilution) or tissue homogenates (starting at a 1:10 dilution) were added, and plates were incubated at room temperature for 2 h. After washing the plates 3 times, horseradish peroxidase (HRP)-conjugated secondary IgG (1:10,000; Abcam, MA, USA), IgA (1:250; Brookwood Biomedical, AL, USA), or IgM (1:250; Brookwood Biomedical, AL, USA) antibodies were added. After addition of secondary IgG antibody, plates were incubated at room temperature for 1 h, while for IgA and IgM antibodies, plates were incubated at 4°C overnight. Sample and antibody dilutions were done in 1% nonfat milk solution in wash buffer. Following washing, reactions were developed by adding 100 μl/well of Sigmafast OPD (*o*-phenylenediamine dihydrochloride) (MilliporeSigma) solution for 10 min and stopped by adding 3 M hydrochloric acid (HCl) solution; plates were read at a 490-nm wavelength using an ELISA plate reader (BioTek Instruments). The endpoint antibody titer was determined by using a cutoff value that is three times the absorbance of the first dilution of mock (uninfected) animal samples.

### Microneutralization assay.

Heat-inactivated (56°C, 35 min) plasma samples were 2-fold serially diluted in infection medium (starting at a 1:20 dilution) and incubated with 100 TCID_50_ of SARS-CoV-2. After a 1-h incubation at room temperature, plasma-virus mix was transferred into a 96-well plate confluent with Vero-E6-TMPRSS2 cells in sextuplet. After 6 h, inocula were removed, fresh infection medium was added, and plates were incubated at 37°C for 2 days. Cells were fixed with 4% formaldehyde and stained with naphthol blue black solution, and neutralizing antibody titer was calculated as described (14).

### Cytokine RT-qPCR.

Flash-frozen hamster lung tissue was sectioned on dry ice and lysed in ice-cold TRIzol using the protein safe hard tissue homogenizing kit (CK28-R, Percellys) in combination with a manufacturer-recommended bead-beating protocol (Percellys Evolution; 8,500 rpm, three 15-s cycles with 30-s breaks on ice after each cycle). TRIzol lysates were immediately processed to harvest total RNA. Total RNA was extracted using the miRNeasy minikit (217004, Qiagen) following the manufacturer’s instructions. RNA concentration and quantity were evaluated using a Nanodrop. Two-step RT-qPCR was performed on a StepOnePlus real-time PCR system (ThermoFisher Scientific) using the high-capacity RNA-to-cDNA kit (4387406, ThermoFisher Scientific) followed by Power SYBR green PCR master mix (436877, Applied Biosystems) and optimized forward and reverse primer pairs (IDT). RT-qPCR sequences for each primer pair used in these studies are listed in Table 1.

**TABLE 1:** RT-qPCR sequences for each primer pair used in this study

Gene name	Forward primer 5′ to 3′	Reverse primer 5′ to 3′	Reference
γ-Actin	ACAGAGAGAAGATGACGCAGATAATG	GCCTGAATGGCCACGTACA	(61)
β-Actin	CCATTGGCAACGAGCGGTT	CCACAGGATTCCATACCCAGGAAG	
IL-1β	TGAGGTTGACGG GCTCCAAAA	ACAGGGGTGTTC CACAGCTT	
TNF-α	TGAGCCATCGTG CCAATG	AGCCCGTCTGCT GGTATCAC	(61)
IL-6	TGTCTTCTTGGGACTGCTGC	CCAAACCTCCGACTT GTTGA	(62)
IFN-α	AGACTGGGAGTTGCCTGTGA	GAGGAATCCAGGGCTTTCCAG	(62)
IFN-β	CCATCATGACCAACAGGTGGA	GTCTGGCCTCAAGTTCCTCG	
IFN-γ	ATGGAGGGGACCTCGTCTTT	GATGGCCTGGTTGTCCTTCA	

### Cytokine ELISAs.

Cytokine concentrations in Triton X-100-inactivated lung and spleen homogenates were determined by individual ELISA kits for hamster IFN-α (https://mybiosource.com; MBS010919), IFN-β (https://mybiosource.com; MBS014227), TNF-α (https://mybiosource.com; MBS046042), IL-1β (https://mybiosource.com; MBS283040), IFN-γ (ARP; EHA0005), IL-10 (ARP; EHA0008), and IL-6 (ARP; EHA0006) as per the manufacturer’s instructions. Samples were prediluted 1:5 to 1:10 as necessary in the appropriate kit sample dilution buffer. Total protein in the homogenates was measured by BCA assay (Thermo Fisher Scientific).

### Statistical analyses.

Statistical analyses were done in GraphPad Prism 9. Changes in body mass were compared using two-way repeated measures analysis of variance (ANOVA) followed by Bonferroni’s multiple-comparison test. Chest CT scores were compared by unpaired Mann-Whitney test. E2 concentrations were compared by two-tailed unpaired *t* test. Virus titers and antibody responses were log transformed and compared using two-way ANOVA or mixed-effects analysis followed by Bonferroni’s multiple-comparison test. Cytokine concentrations were normalized to total protein content in lung homogenates and compared using two-way ANOVA. Associations between cytokines and virus titers in lungs were conducted using Spearman correlational analyses. Differences were considered to be significant at a *P* value of <0.05.

### Data availability.

All data will be made publicly available upon publication and upon request for peer review.
