# Supplementary figures and images for: Sex Differences in Lung Imaging and SARS-CoV-2 Antibody Responses in a COVID-19 Golden Syrian Hamster Model
Source: mBio. 2021 Jul 13;12(4):e00974-21. doi: 10.1128/mBio.00974-21 (PMC8406232; doi:10.1128/mBio.00974-21)

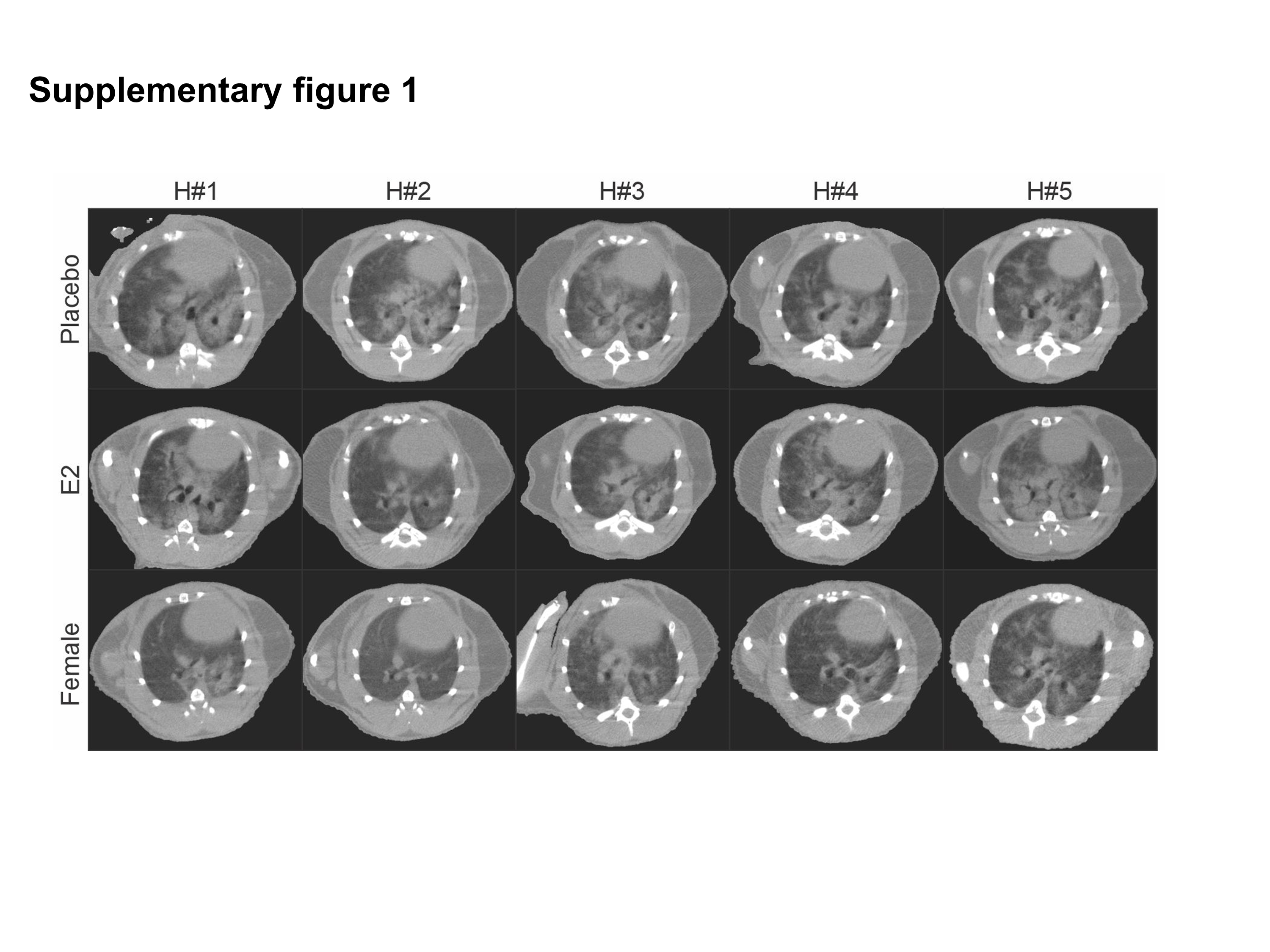

Supplement: FIG S1 [file mbio.00974-21-sf001.tif]

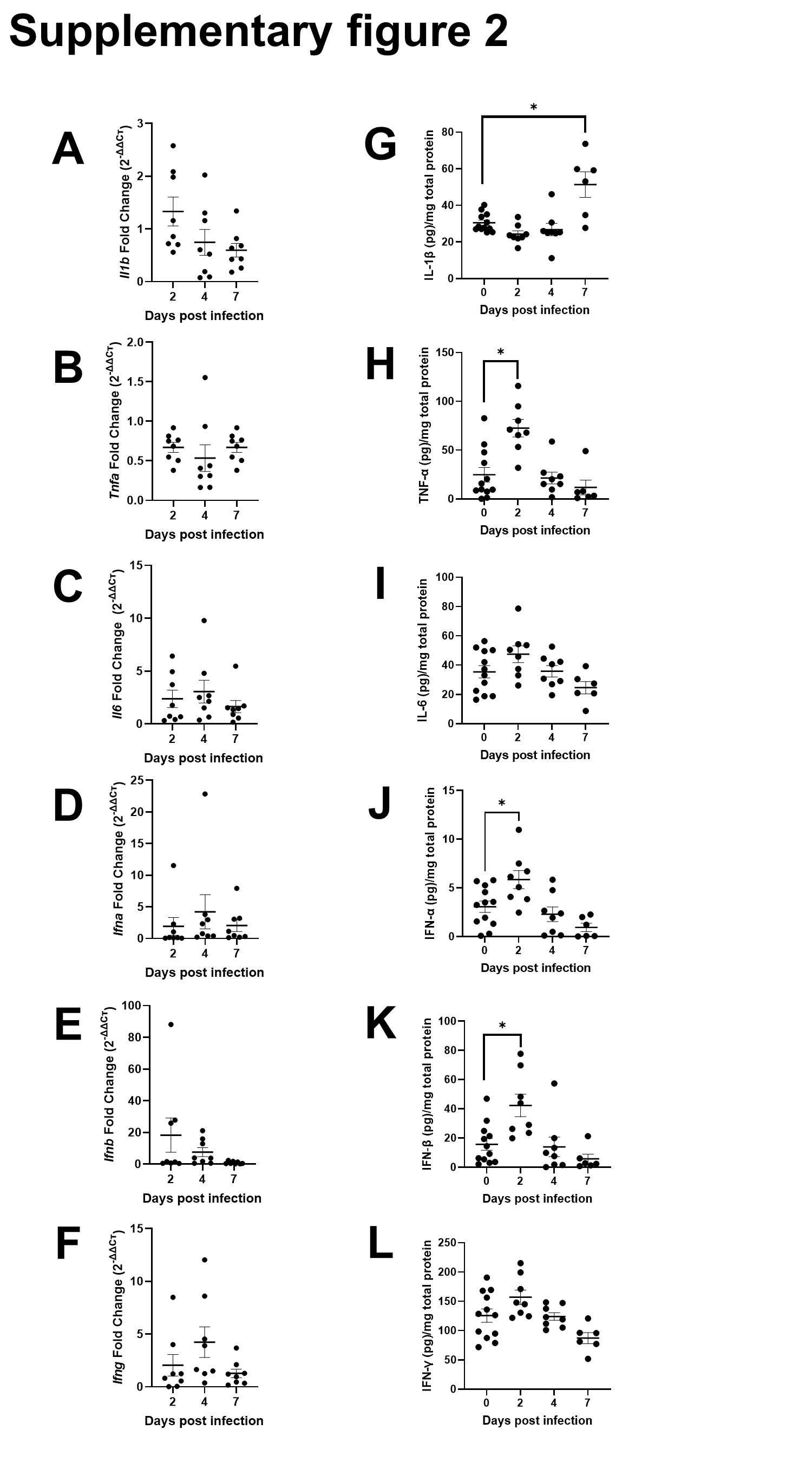

Supplement: FIG S2 [file mbio.00974-21-sf002.tif]
