# Supplementary material for: Sex Differences in Lung Imaging and SARS-CoV-2 Antibody Responses in a COVID-19 Golden Syrian Hamster Model
Source: mBio. 2021 Jul 13;12(4):e00974-21. doi: 10.1128/mBio.00974-21 (PMC8406232; doi:10.1128/mBio.00974-21)
Supplement: TABLE S1 [file mbio.00974-21-st001.pdf]

Supplementary table 1: Concentrations (pg/mg total protein) of cytokines in the lungs and spleen of male and female hamsters at different days post infection (dpi). Mock-infected animal samples from 2, 4, or 7 dpi were pooled and used as 0 dpi. Data are presented as the mean ± the standard error of the mean from one or two independent experiments (n=6-12/group) with no significant differences observed between the groups based on two-way ANOVA (mixed-effects analysis) followed by Bonferroni's multiple comparison test.

**DPI**	**Groups**	**IL-10 Lungs**	**TNF-α Spleen**	**IL-6 Spleen**	**IL-β Spleen**	**IL-10 Spleen**	**IFN-α Spleen**	**IFN-β Spleen**	**IFN-γ Spleen**
0	Male	28.3±4.4	8.5±2.5	30.7±6.8	26.0±10.0	23.2±5.0	3.9±0.6	8.4±3.1	126.3±30.1
	Female	35.9±6.7	6.9±2.5	16.2±4.0	13.6±5.0	12.6±3.3	2.9±0.6	7.4±2.7	106.7±24.6
	Combined	31.8±3.9	7.8±1.7	24.1±4.5	20.4±6.0	18.4±3.4	3.5±0.4	8.0±2.0	117.4±19.1
2	Male	48.6±11.3	13.0±2.7	78.2±50.2	10.1±5.2	65.4±41.2	8.0±1.2	17.3±6.5	277.3±78.2
	Female	43.2±7.2	28.7±8.8	38.6±8.6	10.5±3.0	30.6±7.6	7.1±1.1	27.3±6.7	177.2±42.0
	Combined	45.9±6.3	20.9±5.2	58.4±24.7	10.3±2.8	48±20.5	7.6±0.8	22.3±4.7	227.2±45.2
4	Male	38.5±7.4	31.5±19.1	18.9±1.8	11.2±5.0	862.7±847.7	9.5±5.6	37.5±23.6	222.8±156.3
	Female	34.7±6.9	3.4±1.1	775.5±756.7	6.7±2.7	74.4±61.1	3.8±1.4	3.6±3.2	197.0±110.9
	Combined	36.6±4.7	17.5±10.3	397.2±378.4	8.9±2.8	468.6±420.7	6.6±2.9	20.5±12.7	209.9±88.9
7	Male	25.0±4.6	13.0±9.5	54.6±41.6	5.1±2.1	37.4±27.8	4.3±1.6	14.0±6.4	189.8±95.6
	Female	22.2±6.2	20.5±8.4	38.5±4.8	7.2±1.9	29.9±3.0	6.3±1.2	20.9±5.1	168.4±21.6
	Combined	23.1±4.1	17.3±6.0	45.4±16.3	6.3±1.4	33.1±10.7	5.5±1.0	18.0±3.9	177.6±38.2
